# Enhanced NK-92 Cytotoxicity by CRISPR Genome Engineering Using Cas9 Ribonucleoproteins

**DOI:** 10.3389/fimmu.2020.01008

**Published:** 2020-05-22

**Authors:** Rih-Sheng Huang, Hsin-An Shih, Min-Chi Lai, Yao-Jen Chang, Steven Lin

**Affiliations:** ^1^Institute of Biological Chemistry, Academia Sinica, Taipei, Taiwan; ^2^Institute of Biochemical Sciences, National Taiwan University, Taipei, Taiwan

**Keywords:** CRISPR, Cas9, RNP, nucleofection, NK-92, immunotherapy, ADCC

## Abstract

Natural killer (NK) cells are an attractive cell-type for adoptive immunotherapy, but challenges in preparation of therapeutic primary NK cells restrict patient accessibility to NK cell immunotherapy. NK-92 is a well-characterized human NK cell line that has demonstrated promising anti-cancer activities in clinical trials. Unlimited proliferation of NK-92 cells provides a consistent supply of cells for the administration and development of NK cell immunotherapy. However, the clinical efficacy of NK-92 cells has not reached its full potential due to reduced immune functions as compared to primary NK cells. Improvements of NK-92 functions currently rely on conventional transgene delivery by mRNA, plasmid and viral vector with limited efficiencies. To enable precise genetic modifications, we have established a robust CRISPR genome engineering platform for NK-92 based on the nucleofection of Cas9 ribonucleoprotein. To demonstrate the versatility of the platform, we have performed cell-based screening of Cas9 guide RNA, multiplex gene knockout of activating and inhibitory receptors, knock-in of a fluorescent gene, and promoter insertion to reactivate endogenous CD16 and DNAM-1. The CRISPR-engineered NK-92 demonstrated markedly enhanced cytotoxicity and could mediate antibody-dependent cellular cytotoxicity against hard to kill cancer cell lines. Our genome editing platform is straightforward and robust for both functional studies and therapeutic engineering of NK-92 cells.

## Introduction

Natural killer (NK) cells are potent innate effectors capable of targeting and killing virally infected and malignant cells (1). Unlike T cells, NK cells do not require a matching human leukocyte antigen for activation or functions. Instead, NK cells rely on an array of germline-encoded activating and inhibitory receptors that engage their cognate ligands on the target cells and initiate cytotoxicity (2). NK cells not only secrete granzyme B and perforin that lyse the target cells, but also cytokines and chemokines to orchestrate the subsequent immune responses (2). These unique attributes make NK cells an attractive cell type for adoptive immunotherapy.

Evidence from clinical studies demonstrates that NK cell immunotherapy is effective and safe (3–8), but there are still challenges, particularly in the manufacture of therapeutic NK cells. Because NK cells represent only 10% of peripheral lymphocytes, the supply of NK cells from leukapheresis is limited (9). *Ex vivo* expansion is necessary to generate clinically relevant levels of primary NK cells for infusion; however, this process is complicated by telomere shortening and reduced cytotoxicity of the resulting cells (9). Although allogeneic transfer of NK cells is safe, depletion of contaminating allogeneic T cells is crucial to prevent graft-vs.-host reaction. The logistics and costs associated with the preparation of primary NK cells have restricted NK cell immunotherapy to highly selected patients (9).

To overcome the limitations of primary NK cells, several clonal NK cell lines were established from patients with NK-cell lymphoma (10). Among them, NK-92 cell line has shown consistent anti-cancer activities in several clinical studies (10). NK-92 cells possess many hallmark activating receptors (for example, NKG2D, NKp30, NKp44, and NKp46), and yet lack several inhibitory receptors (for example, TIGIT and PD-1) (11). Infusion of gamma-irradiated NK-92 cells has also been proven safe to patients (9, 12). Furthermore, unlimited proliferation of NK-92 generates a consistent supply of homogeneous NK cells to allow multiple infusions, improve the logistics of treatment and reduce the cost for therapeutic development (9). However, NK-92 has reduced anti-cancer activities when compared to primary NK cells due to compromised immune functions. A better understanding of NK-92 immunobiology is vital to increase the efficacy of NK-92 adoptive immunotherapy, and efficient genetic toolkits are required to achieve that goal.

Recent advances in genome editing technologies such as CRISPR has reinvigorated interests in NK cell genetic engineering. However, genetic modifications of NK-92 still rely largely on transgene delivery by mRNA, plasmid DNA and viral vectors (13–17). These conventional methods are confined by transient expression, low transfection efficiency, inconsistent transduction, and random genomic integration of vector DNA. A more robust and precise method is needed for next generation NK-92 engineering. Recently, nucleofection of pre-assembled CRISPR-Cas9 ribonucleoprotein (Cas9 RNP) has shown promising genome editing efficiencies in primary NK cells (18–20). We sought to explore this approach for NK-92 genome engineering, only to discover that the conditions for primary NK cells were not transferable to NK-92.

Here we describe a powerful and versatile Cas9 RNP-based genome editing platform for NK-92 cells. We have used this platform for cell-based screening of Cas9 single guide RNA (sgRNA). We also demonstrated that multiplex KO of activating and inhibitory receptors was effective and viable, as opposed to plasmid-based CRISPR editing. Finally, we performed Cas9-mediated homology-directed repair (HDR) to insert restriction sites, a fluorescent gene, and also a synthetic promoter to reactivate silenced endogenous genes. The CRISPR-engineered NK-92 cells were enriched and expanded to demonstrate significantly enhanced cytotoxicity against cancer cell lines. This work represents the first reliable gene editing method for this clinically important NK cell line.

## Materials and Methods

### Cell Culture

All cell culture reagents and media were purchased from Gibco (Thermo Fisher Scientific) unless stated otherwise. All cell lines were of human origin, maintained in 37°C incubator with 5% CO_2_ in specific media, and routinely tested for mycoplasma contamination by EZ-PCR detection assay kit (Biological Industries). The malignant non-Hodgkin's lymphoma cell line NK-92 (ATCC) were maintained in RPMI 1640 Medium (ATCC modification) supplemented with 15% heat-inactivated fetal bovine serum (FBS), 25 mM HEPES, 1X GlutaMAX, 1X Antibiotic-Antimycotic, and 100 U/ml IL-2 (PeproTech). NK-92 cells were passaged every 2–3 days to maintain the cell density at 2 × 10^5^ – 8 × 10^5^ cells/ml. The Burkitt's lymphoma cell line Raji was maintained in RPMI 1640 Medium (ATCC modification) supplemented with 15% heat-inactivated FBS, 25 mM HEPES, 1X GlutaMAX and 1X Antibiotic-Antimycotic. The adenocarcinoma cell line HeLa (ATCC), the embryonic kidney cell line HEK293T (ATCC), the adenocarcinoma cell line MDA-MB-231 (gift from Dr. Ruey-Hwa Chen in Academia Sinica), and the ductal carcinoma cell line BT-474 (BCRC, Taiwan) were maintained in DMEM with high glucose (HyClone) supplemented with 15% heat-inactivated FBS, 25 mM HEPES, 1X GlutaMAX and 1X Antibiotic-Antimycotic.

### Flow Cytometry and Fluorescence-Activated Cell Sorting

The following antibodies were used for flow cytometry and fluorescence-activated cell sorting (FACS): APC anti-CD96 (NK92.39, BioLegend), BV-421 anti-CD96 (NK92.39, BioLegend), PE anti-NKG2A (REA110, Miltenyi Biotec), PE anti-DNAM-1 (11A8, BioLegend), APC anti-NKp46 (9E02, BioLegend), APC anti-NKG2D (1D11, BioLegend), PerCP-Cy5.5 anti-CD16 (3G8, BioLegend), APC Mouse IgG1κ Isotype (MOPC-21, BioLegend), BV-421 Mouse IgG1κ Isotype (MOPC-21, BioLegend), and PE REA control (REA293, Miltenyi Biotec). All the experiments were performed in CytoFLEX (Beckman Coulter), FACSJazz or FACSAria IIIu (BD Biosciences). The data were analyzed with FlowJo (BD Biosciences) and CytExpert (Beckman Coulter). Ice-cold FACS buffer (DPBS supplemented with 2% FBS, 25 mM HEPES and 0.5 mM EDTA) was used for washing, cell resuspension and antibody dilution. Briefly, the cells were pelleted by centrifugation at 300 g for 5 min, washed once and stained in the antibody solution (diluted as per manufacturer's recommended ratios) in the dark for 20 min on ice. After staining, the cells were washed once, resuspended and kept on ice before analysis. After FACS, the enriched cells were pelleted at 300 g for 5 min, resuspended to 2 × 10^5^ cells/ml in NK-92 culture medium and proceed to standard culture method.

### Viability Assays by Zombie Dye and Precision Beads

Zombie Violet Fixable Viability Kit, Precision cell count beads and 7-Aminoactinomycin D (7-AAD) were purchased from BioLegend. In Zombie dye assay, cells were pelleted at 300 g for 5 min and followed by DPBS wash once. The cells were then stained in 1,000-fold diluted Zombie dye in the dark for 20 min, washed once and resuspended in FACS buffer for analysis. In Precision beads assay, cells were gently resuspended by pipetting, and filtered through 35-μm nylon mesh cell strainer (Corning). Precision beads were resuspended thoroughly before use by vortexing for 40 s. Precision beads solution was added at 0.1 v/v ratio to each filtered cell sample and vortexed at low speed for 5 s. The samples were then stored on ice until analysis by flow cytometry. One thousand Precision beads were counted, by APC and PB450 signals, to serve as an internal standard to quantitate the cell density (gated as P1 and shown as the red rectangle in Figure S1). 7-AAD staining and an FSC/SSC scattering plot were used to set an electronic gate on viable cells (gated as P2 and shown as the black circle in Figure S1). The following equations were used to calculate the percentages of recovery of viable cells and viable GFP^+^ cells.

$$\begin{array}{l} \text{Recovery of viable cells }\left(\text{\%}\right)\;=\;\frac{\text{Counts of viable cells in the sample}}{\text{Counts of viable cells in the untreated control}}\\ \;\times 100 \end{array}$$

$$\begin{array}{l} \text{Viable GF}{\text{P}}^{+}\text{ cells }\left(\text{\%}\right)\;=\;\frac{\text{Counts of GF}{\text{P}}^{+}\text{ cells in the viable cells}}{\text{Total viable cells}}\\ \;\times \;100 \end{array}$$

### Preparation of Cas9 Protein and sgRNA

Cas9 recombinant protein was over-expressed in *E. coli* BL21 (DE3) from plasmid pMJ915 (Addgene # 69090), and purified as described previously (21). Cas9 protein was stored at −80°C in Cas9 RNP buffer (20 mM HEPES at pH 7.5, 150 mM KCl, 10% glycerol and 1 mM β-mercaptoethanol). The sgRNAs were designed by the CRISPR Design tool on Benchling website (www.benchling.com) that provided predictions for on-target efficiency and off-target effect. The sgRNAs with high off-target scores (indicating high editing precision and low off-target effect) were selected and synthesized by *in vitro* transcription (IVT) using T7 RNA polymerase as described previously (22). The DNA oligonucleotides for IVT template assembly were listed in Table S2. The synthesized sgRNAs were purified by denaturing urea-PAGE. The RNA bands corresponding the full-length sgRNA were excised to remove truncated forms. Additionally, the PAGE-purified sgRNAs were treated with calf-intestine phosphatase to remove the 5′ phosphate group to prevent triggering innate immune responses (23). The final sgRNA products were dissolved in Cas9 RNP buffer, quantitated by NanoDrop Lite (Thermo Fischer Scientific) and stored as aliquots at −80°C.

### Cas9 RNP Nucleofection

Cas9 RNP complexes were assembled immediately before nucleofection, by mixing equal volumes of 40 μM of Cas9 protein and 48 μM of sgRNA at molar ratio of 1:1.2 and incubating at 37°C for 15 min. The final concentration of Cas9 RNP was defined as 20 μM. An nucleofection reaction consisted of 4 × 10^5^ of NK-92 cells in 20 μl of nucleofection buffer, 2 μl of Cas9 RNP (equivalent to 40 pmol) and 2 μl of HDR DNA at the indicated concentration. In RNP dosage experiment, 4 and 6 μl of Cas9 RNP were added to obtain 80 and 120 pmol. The nucleofection buffer was either P3 (Lonza) or Sol2, which was composed of 150 mM sodium phosphate buffer (pH 7.2), 5 mM KCl, 15 mM MgCl_2_, 15 mM HEPES and 50 mM mannitol (24). Sol2 was stored at 4°C and replaced every month. Freezing of Sol2 is not recommended due to precipitation. The nucleofection mixtures were then transferred into 16-well strip for nucleofection in Lonza 4D Nucleofector using the buffer and pulse code specified. Pipetting must be careful to prevent air bubbles trapped between the electrodes. Immediately after nucleofection, 100 μl of pre-warm NK-92 culture medium was added to each well for cell recovery in 37°C incubator for 15 min. The cells were then transferred to the culture plate filled with pre-warm culture medium. All analyses were performed 72 h after nucleofection unless otherwise stated.

### Gene Editing Analyses by DNA Sequencing

The cells were pelleted at 300 g for 5 min and washed with DPBS once. Genomic DNA was extracted by lysing the cell pellet in QuickExtraction solution (Lucigen) at 65°C for 15 min and then 98°C for 5 min. The extracted genomic DNA was stored at −20°C. PCR amplification of the target sequences was performed using KAPA HiFi HotStart PCR kit. The primer sequences and PCR conditions were listed in Table S3. The PCR products were purified by QIAquick PCR Purification Kit (Qiagen) and eluted in molecular-grade water. Fifty microgram of PCR DNA was used for Sanger Sequencing. The percentages of indel and HDR were analyzed online by Inference of CRISPR Edits (ICE) tool (https://www.synthego.com/products/bioinformatics/crispr-analysis).

### Deep Sequencing Analysis of On-Target and Off-Target Sites

Off-target sites were predicted by the CRISPR Design tool on the Benchling website (www.benchling.com) based on the published algorism (25). The genomic sequences of the on-target site and two of the top predicted off-target sites were PCR amplified by the primer sets and conditions (Table S3). Briefly, target amplicons were amplified by 30 cycles of PCR from 300 ng of genomic DNA in QuickExtraction solution (Lucigen) using KAPA HiFi HotStart DNA Polymerase kit (KAPA Biosystems). The PCR amplicons were purified with Qiagen Gel Purification Spin Column, and subjected to QC assessment with Qubit DNA quantification (Thermo) and size profiling using Fragment Analyzer (Agilent). To add the dual-barcoded adaptor to the amplicons, Nextera XT Index Kit v2 (Illumina) was applied for indexing PCR with 5 μl of the amplicon template in 50 μl reactions, and amplified for 8 cycles using 2X KAPA HiFi Mastermix (KAPA Biosystems). The PCR products were cleaned up by AmPure beads (Beckman Coulter), and subjected to QC with Qubit and Fragment Analyzer as well as qPCR for molar concentration normalization using Kapa Illumina Library Quantification Kit (KAPA Biosystems) prior to library pooling. High throughput sequencing of PE2^*^151 bp was carried out on a MiSeq sequencer (Illumina), and obtained a total of 19.41 millions of pass-filter clusters at PF of 84.6% and >Q30 bases at 96 and 92% for Read1 and Read2, respectively. The dataset was generated and demultiplxed with BclToFastq 2.18 pipeline (lllumina). FASTQ reads were first processed by 30-bp HEADCROP trimming using Trimmomatic to eliminate the low-quality bases at the 5′ and 3′ ends. After trimming, the files were analyzed by CRISPresso2 against human reference genome GRCh38 with default parameters. Random single nucleotide substitutions were discarded as amplification and sequencing errors. Deep sequencing data is available at the NCBI Sequence Read Archive (PRJNA608597). The % Indel of on-target and off-target sites were calculated by the following equation:

$$\begin{array}{l} \text{\% Indel }=\;\frac{\text{Number of insertion reads}+\text{number of deletion reads}}{\text{Total number of reads}-\text{number of substitution reads}}\\ \;\times \;100 \end{array}$$

We detected a single-nucleotide variant in *KLRK1* on-target region in 97% of the reads. We didn't filter out this single nucleotide substitution. The following equation was used for *KLRK1* calculation:

$$\begin{array}{l} \text{\% Indel }=\;\frac{\text{Number of insertion reads}+\text{number of deletion reads}}{\text{Total number of reads}}\\ \;\times \;100 \end{array}$$

### Chromosomal Translocation Assay

The triple-negative population of edited NK-92 cells was isolated by FACS, and the genomic DNA was extracted by QuickExtraction solution (Lucigen). Chromosomal translocation was detected by an end-point PCR assay as described previously (26). Genomic amplification was performed using KAPA HiFi HotStart DNA Polymerase kit (KAPA Biosystems) and 15 combinations of forward and reverse primers for *CD96, KLRC1* and *NCR1* target loci (Figure S2A and Table S3). The thermocycler setting consisted of 30 cycles of 98°C for 10 s, 65°C for 10 s, and 72°C for 20 s, except for the combinations with *CD96* forward primer, which consisted of 30 cycles of 98°C for 10 sec, 70°C for 10 s and 72°C for 20 s. The DNA products were resolved in 2% agarose gel in TAE buffer and post-stained with SYBR Safe (Thermo Fischer Scientific) for visualization.

### Construction and Preparation of HDR Templates

The HDR templates were constructed by Gibson Assembly using NEBuilder HiFi DNA Assembly kit (NEB). The constructs were composed of DNA fragments as described below, and were cloned into Sph1-BamH1 double-digested pUC19 vector. Left homologous arm of *CD96* (Fragment 1), right homologous arms of *CD96* (Fragment 2) and *mCherry* gene (Fragment 3) were assembled to CD96-mCherry HDR template. Left homologous arm of *FCGR3A* (Fragment 4), right homologous arms of *FCGR3A* (Fragment 5) and SFFV promoter (Fragment 6) were assembled to SFFV-CD16 HDR template. Left homologous arm of *CD226* (Fragment 7), right homologous arms of *CD226* (Fragment 8), and SFFV promoter (Fragment 9) were assembled into SFFV-CD226 HDR template. Fragment 1, 2, 4, 5, 7, and 8 were PCR amplified from NK-92 genomic DNA. Fragment 3 was amplified from pTR144 (Addgene # 112013). Fragment 6 and 9 were amplified from LeGO-iT2 (Addgene # 27343). Fragment PCR was performed using KAPA HiFi HotStart DNA Polymerase kit (KAPA Biosystems).

To prevent targeting of HDR template by Cas9 RNP, mutations were introduced into the HDR templates by Round-the-horn site-directed mutagenesis. In the *FCGR3A* template, the sgRNA16 PAM sequence in the left homology arm was mutated from AGG to ATT. In the *CD226* template, the sgRNA22 seed region sequence in the right homology arm was modified to silent mutations. Illustrations of *mCherry, FCGR3A*, and *CD226* HDR templates are in Figures S3–S5, respectively. The PCR conditions and the primer sequences are listed in Table S3. All constructs were validated by Sanger sequencing at the DNA sequencing core facility at the Institute of Biomedical Sciences at Academia Sinica. For restriction sequences KI experiments, the ssDNA ultramers were purchased from IDT DNA. Complete HDR template sequences are provided in Table S4.

For HDR experiments, linear dsDNA PCR templates were amplified from the plasmid constructs using KAPA HiFi DNA Polymerase kit (KAPA Biosystems) and the primers listed in Table S3. The PCR reaction mixture was purified by AMpure XP beads (Beckman Coulter) at 0.7 v/v ratio as per manufacturer's protocol. The dsDNA was eluted in molecular H_2_O and precipitated by isopropanol at −20°C overnight. The precipitated dsDNA pellet was then washed three times by 70% ethanol, dried under vacuum and resuspended in molecular H_2_O. The concentration of dsDNA was determined by NanoDrop Lite, adjusted to 1 μg/μl and stored at −20°C.

### Construction of Cas9:sgRNA Dual Expressing Plasmids

The sgRNA guide sequences were inserted into a modified version of pX330 (Addgene # 42230), carrying an extended sgRNA scaffold for improved activity (5′ GTTTAAGAGCTATGCTGGAAACAGCATAGCAAGTTTAAATAAGGCTAGTCCGTTATCAACTTGAAAAAGTGGCACCGAGTCGGTGCTTTTTT 3′). The sgRNA cloning method was as described previously (27). Briefly, complementary ssDNA oligonucleotides, which encoded the guide sequences, were purchased from IDT DNA and annealed to create dsDNA with overhangs. The overhangs directed the ligation of annealed dsDNA with BbsI-digested pX330 vector, inserting the guide sequences downstream of U6 promoter sequence for expression. The sequences of complementary DNA oligonucleotides were as follow. For *CD96* targeting: 5′ CACCGTGCAGATGCAATGGTCCA 3′ and 5′ AAACTGGACCATTGCATCTGCAC 3′. For *TIGIT* targeting: 5′ CACCGCCTCCTGATCTGGGCCCAG 3′ and 5′ AAACCTGGGCCCAGATCAGGAGGC 3′. For *KLRC1* targeting: 5′ CACCGAACAGGAAATAACCTATG 3′ and 5′ AAACCATAGGTTATTTCCTGTTC 3′. BbsI digestion and T4 ligation were performed according the manufacturer's protocol (NEB). The plasmids were validated by Sanger sequencing.

### Plasmid DNA Nucleofection

NK-92 cells were prepared as described above. Four hundred ng of pmaxGFP (Lonza) per 4 × 10^5^ of cells was used for condition screening. GFP expression and cell viability were analyzed by flow cytometry at 4 and 24 h after nucleofection. For plasmid-based gene editing, Cas9:sgRNA dual expression plasmids were purified by Plasmid Midi Kit (Qiagen) and 2 μg of DNA was used per nucleofection. The reduction in target protein expression was analyzed by immunostaining and flow cytometry at 72 h after nucleofection. For HDR experiments, 100 pmol of ssDNA ultramer or 2 μg of dsDNA PCR template was used per nucleofection.

### Calcein-AM Cytotoxic Assay

Trypsin should not be used for cell dissociation in the cytotoxicity assay to prevent digestion of NK cell-targeting ligands. Adherent cells were detached by enzyme-free cell dissociation buffer (Gibco), neutralized by culture medium and pelleted at 200 g for 3 min. The supernatant was aspired and the target cells were wash by DPBS. After washing, 1 × 10^6^ cells were resuspended in 1 ml DPBS containing 10 μM Calcein-AM (BioLegend) and incubated at 37°C for 30 min. The target cells were then washed by culture media for three times and resuspended at the cell density of 8 × 10^5^ or 1.6 × 10^6^ cells/ml in RPMI-1640 (ATCC modification). NK-92 cells were pelleted at 90 g for 10 min and resuspended at the cell density of 1 × 10^5^ cells/ml in RPMI-1640 (ATCC modification). One hundred microliter of NK-92 cells per well were added to U-bottom 96-well plates and serial dilution was performed for different NK-92-to-target ratios. One hundred microliter of the stained target cells were directly added into each well. For ADCC assay, BT-474 and Raji cells were incubated with Herceptin (Trastuzumab, Roche) and Rituxan (Rituximab, Roche), respectively, for 30 min in 37°C incubator prior to being added into each well. The 96-well plate was the centrifuged at 120 g for 1 min to accelerate the contact between NK-92 and target cells. The cells were allowed to co-culture for 4 h at 37°C incubator. Spontaneous release of Calcein-AM was measured in the absence of NK-92 cells. Maximal release was determined by complete lysis of target cells in RPMI-1640 (ATCC modification) containing 2% Triton-X100. After co-culture, the plates were centrifuged at 120 g for 1 min, and 100 μl of the supernatant was transferred to 96-well Opti-plates (PerkinElmer). The 488/520 values were recorded by M1000 pro (Tecan). The following equation was used for cytotoxicity calculation:

$$\begin{array}{l} \text{Target lysis }\left(\text{\%}\right)=\;\frac{\text{Experimental release}-\text{spontaneous release}}{\text{maximal release}-\text{spontaneous release}}\\ \;\times \;100 \end{array}$$

### Statistical Analyses

Except for the screening experiments, all data were collected from three independent experiments to determine mean values ± SD as shown. Two-tailed Welch's unequal variances *t*-test was used to test for significant differences between two groups. *P*-values ≤ 0.05 were considered statistically significant. Statistical analyses were performed using GraphPad Prism 8.

## Results

### Gene KO by Cas9 RNP Nucleofection Is Efficient and Viable

**Figure 1:**
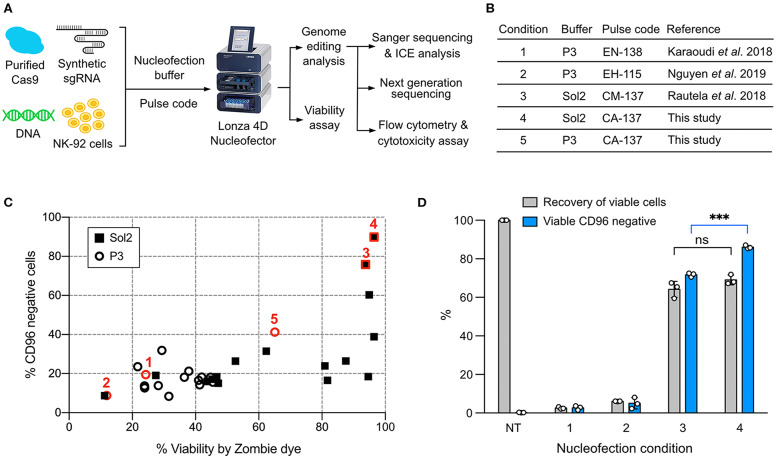
Screening of the nucleofection conditions for Cas9 ribonucleoprotein (RNP) delivery. **(A)** Workflow of NK-92 genome editing by Cas9 RNP and DNA nucleofection using a Lonza 4D Nucleofector system. **(B)** A list of the reference and newly identified nucleofection conditions. **(C)** Sixteen pulse codes in combination with two buffers, Sol2 and P3, were tested to deliver Cas9 RNP to knockout (KO) *CD96* gene. Conditions 1–5 are highlighted in red to show their relative *CD96* KO efficiencies and cell viability. **(D)** Conditions 1–4 were re-examined by a more accurate viability assay. Recovery of viable cells was determined by Precision beads assay and normalized to untreated cells (NT). CD96^−^ cells in the viable population were quantitated. Data are shown as mean ± SD of three independent experiments. Statistics by two-tailed Welch's unequal variances *t*-test; ns, not significant and ***p ≤ 0.001. The pulse codes, buffers and raw data are summarized in Table S1.

We used a Lonza 4D Nucleofector for NK-92 nucleofection because of its non-toxic carbon-based electrodes and semi high-throughput and scalable capability. However, the trouble is that Lonza nucleofection solutions and pulse codes are propriety, and condition screening is therefore necessary to optimize payload delivery. To enable robust gene knockout (KO) and knock-in (KI) in NK-92, we identified a combination of nucleofection buffer and pulse code for optimal co-delivery of Cas9 RNP and DNA repair template. The genotype and phenotype of genome edited NK-92 were assessed by Sanger sequencing and Inference of CRISPR Edit (ICE) tool, next generation sequencing (NGS), flow cytometry and cytotoxicity assay (Figure 1A).

To screen for Cas9 RNP nucleofection conditions, we programmed Cas9 to target *CD96* gene, encoding a highly expressed NK cell inhibitory receptor. Cas9 cleavage at *CD96* triggers DNA double-stranded break repair by predominantly the error-prone Non-homologous End Joining (NHEJ) pathway (28). NHEJ frequently results in random insertion or deletion (indel), leading to frame-shift mutation and premature termination of protein synthesis. The reduction in CD96 expression was analyzed by flow cytometry as the phenotypic readout of gene KO efficiency. Cell viability was simultaneously monitored by Zombie dye.

We built upon the reference conditions from primary NK cells and performed new rounds of screening. We tested 16 pulse codes in two different nucleofection buffers: Lonza P3 and Solution2+mannitol (Sol2). Sol2 was originally formulated for T cells and later adopted for primary NK cells (19, 24). The 32 combinations contained three reference conditions for primary NK cells: (1) Lonza P3 with EN-138 pulse code (20), (2) P3 with EH-115 (18), and (3) Sol2 with CM137 (19) (Figure 1B). For parallel comparison, we standardized the NK-92 cell density (4 × 10^5^ cells) and RNP concentration (40 pmol). Overall, Sol2 performed significantly better than P3, with condition 3 producing higher *CD96* KO efficiency and cell viability than conditions 1 and 2 (Figure 1C). These results were unexpected, because condition 1 yielded ~75% KO at *TGFBR2* gene in the original report (20). Condition 2 was described for KI of *GFP* gene, but no KO efficiency was mentioned (18). We also identified a new condition 4 (Sol2 with CA-137) that further improved the KO efficiency to ~90% while preserving similar viability (Figure 1C). The pulse codes, buffers, and raw data are summarized in Table S1.

### Precision Cell Count Beads Improves the Accuracy of Viability Assay

The recovery of viable and gene-edited cells is also an important consideration. In some conditions, we noticed substantial cell debris after nucleofection, which was difficult to collect by centrifugation. This led to the loss of dead cells and overestimation of cell viability. To improve the accuracy of the viability assay, we adopted a flow cytometric method to calculate cell density using Precision cell count beads and to determine viable cells by light scattering and 7-Aminoactinomycin D (7-AAD) counterstaining (Figure S1A). This method eliminated the need for centrifugation and cell washing altogether and allowed retention of both the live cells and dead debris. Viable cells were 7-AAD negative and displayed normal dimensions as the untreated cells (black circle, Figure S1B).

We re-examined conditions 1–4 using this more rigorous viability assay. Both conditions 3 and 4 outperformed conditions 1 and 2, confirming that Sol2 was better than P3 in maintaining viable NK-92 cells (Figure 1D). Condition 3 and 4 had a similar recovery of viable cells, but within the viable population, condition 4 produced more CD96^−^ cells (86 ± 0.9%) than condition 3 (71.7 ± 1.1%). The results show that high KO efficiency and cell viability can be achieved by Cas9 RNP nucleofection, but that NK-92 requires specific nucleofection conditions different from primary NK cells.

### High Cas9 RNP Dosage Maintains Targeting Precision

**Figure 2:**
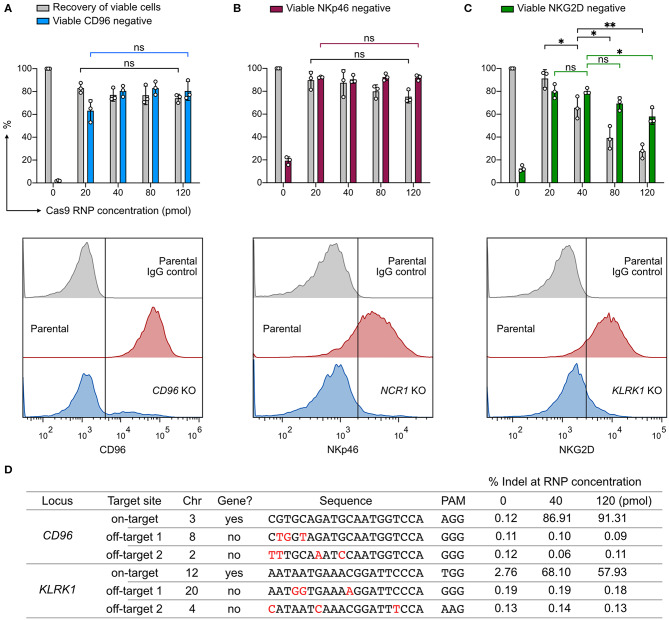
Effect of Cas9 RNP dosage to KO efficiency, cell viability, and off-target cleavage. **(A)** Recovery of viable cells and viable CD96^−^ cells were determined at increasing dosages of Cas9 RNP by Precision beads assay. Representative flow cytometry plots using 40 pmol of Cas9 RNP are shown with the mean percentages of viable negative cells. **(B)** KO results of *NCR1*, which encodes the NKp46 receptor. **(C)** KO results of *KLRK1*, which encodes the NKG2D receptor. **(D)** Analysis of *CD96* and *KLRK1* on-target and off-target editing by next generation sequencing. Mismatches between the on- and off-target sequences are labeled in red. Sequence variations were determined by CRISPResso2 and presented as % indel. Data are shown as mean ± SD of three independent experiments. Statistics by two-tailed Welch's unequal variances *t*-test; ns, not significant, *p ≤ 0.05, **p ≤ 0.01.

We wanted to know whether Cas9 RNP concentration would impact KO efficiency, recovery of viable cells, or off-target cleavage. To probe this, we nucleofected increasing concentrations of *CD96*-targeting RNP ranging from 20 to 120 pmol. Higher concentrations of RNP (80 and 120 pmol) did not increase the *CD96* KO efficiency beyond that of 40 pmol RNP (Figure 2A). At 20 pmol, we observed a slight decrease in KO efficiency to 63%, but the difference was not statistically significant (*P* = 0.07). Similarly, no significant improvement in KO efficiency was observed at *NCR1* (Figure 2B). In both *CD96* and *NCR1* experiments, the recovery of viable cells remained high, at ~70–90% across the RNP concentrations. The outcome of *KLRK1* KO was different (Figure 2C). This gene encodes the NKG2D activating receptor that is important for NK cell proliferation and survival (29). Increasing the RNP dosage led to the reduction in viable cells that was also accompanied by decreasing KO efficiency. Our results indicate that *KLRK1* KO cells cannot survive, and suggest essential roles for NKG2D in NK-92.

To analyze off-target effects, we focused on the *CD96*- and *KLRK1*-targeting sgRNAs, and selected the top two off-target sites as predicted by the CRISPR Design tool on the Benchling website. This web tool provides *in silico* prediction of on-target (editing efficiency) and off-target (editing precision) scores using the algorithms developed by Doench et al., and Hsu et al., respectively (25, 30). All sgRNAs in this work were designed using the same tool and selected for high off-target scores to ensure targeting precision. As a result, we did not detect any off-target mutation at frequencies higher than the untreated control at 40 or 120 pmol of RNP (Figure 2D). ICE and NGS analyses also revealed comparable on-target editing efficiencies. We observed a single thymidine indel at 2.76% frequency in the *KLRK1* on-target region at chromosome 12 position 10379779, even in the untreated cells. This thymidine indel happened in a poly-T track and was likely due to sequencing error. The off-target analysis shows that, with careful sgRNA design, genome editing by RNP nucleofection has high on-target efficiency and no detectable off-target cleavage at the predicted sites at up to 120 pmol of RNP. The dosage experiment suggests that 20–40 pmol of Cas9 RNP per 4 × 10^5^ cells is an effective dosage for robust and precise KO in NK-92 cells.

### *In silico* Prediction Does Not Guarantee sgRNA Performance in Cells

**Figure 3:**
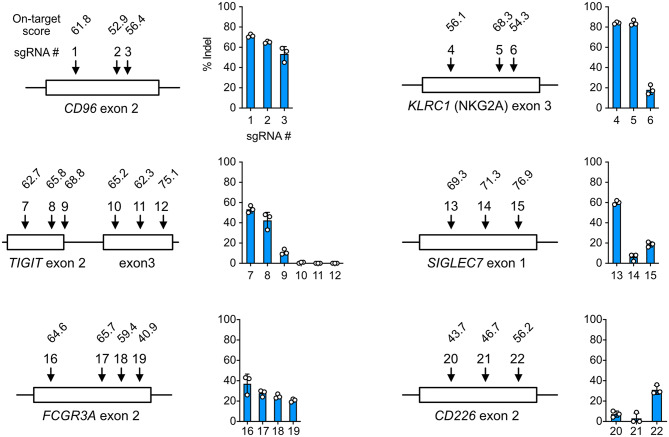
Cell-based screening of efficient sgRNA. sgRNAs (22 in total) were synthesized to target the *CD96, KLRC1, TIGIT, SIGLEC7, FCGR3A*, and *CD226* genes at the indicated exons. On-target scores of the sgRNAs were predicted *in silico* by the CRISPR Design tool on the Benchling website, and are indicated above the sgRNA number. A higher on-target score predicts higher editing efficiency. Percentages of insertion-or-deletion (indel) were determined by Sanger sequencing and ICE analysis. Data are shown as mean ± SD of three independent experiments. The sgRNA position and exon length are relative and not to scale.

Gene KO is a popular approach to delineate gene functions, and Cas9 RNP nucleofection is a rapid platform to identify robust sgRNAs and accessible target regions. To demonstrate this, we tested a series of sgRNAs and discovered that the performance of sgRNAs in the cells did not always agree with the *in silico* prediction of on-target score by Benchling CRISPR Design. For example, sgRNA4 and 6 of the *KLRC1* locus had similar on-target scores, but their editing efficiencies differed by more than 4-fold at 84 ± 3% and 18 ± 5%, respectively (Figure 3). At the *TIGIT* locus, exon 3 appeared inaccessible to Cas9 targeting regardless of on-target scores. Several other examples are shown in Figure 3. These data underscore the importance to experimentally validate sgRNAs in the target cells. Using Cas9 RNP nucleofection, we could quickly test sgRNA performance in NK-92 cells and identify the suitable sgRNA for KO and KI experiments.

### Cas9 RNP Nucleofection Enables Robust Multiplex KO

Knocking out multiple genes one at a time is laborious and time-consuming. Multiplex KO is more straightforward to set up using the Cas9 RNP platform than the plasmid approach, which is restricted by the available copies of sgRNA expression cassette and cloning site on the plasmid. Cas9 RNPs of distinct targeting specificities can be pooled together *in vitro* at precise molar ratios for nucleofection. This approach eliminates repeated KO procedure and allows simultaneous disruption of multiple genes to study the combinatorial effect. To demonstrate this, we combined the best sgRNAs in a single nucleofection reaction for double and triple KO of NK-92 cell surface receptors.

**Figure 4:**
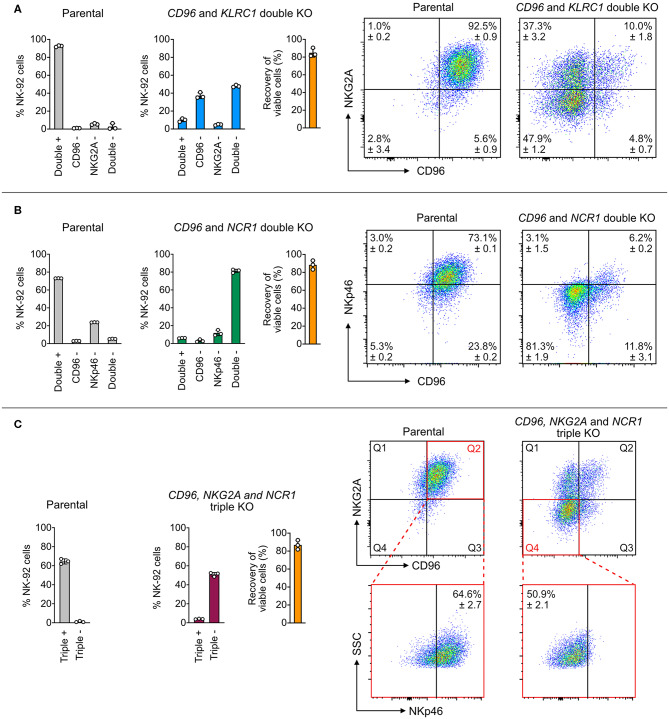
Double and triple KO in a single nucleofection. **(A)** A mixture of two Cas9 RNPs, consisting of sgRNAs targeting both *CD96* and *NKG2A*, was nucleofected into NK-92 cells to simultaneously KO *CD96* and *NKG2A*. Representative flow cytometry plots show the expression levels of target proteins in the parental and double KO cells. **(B)** Double KO of *CD96* and *NCR1* (encoding NKp46). **(C)** Triple KO of *CD96, NKG2A*, and *NCR1* by nucleofection of a mixture of three Cas9 RNPs. To increase readability, only the percentages of triple positive and negative cells are shown in the bar graph. In the representative flow cytometry plots, Q2 of the parental plot was gated to determine triple positive cells. Q4 of the triple KO cells was gated to determine triple negative cells. Cell viability was determined by the Precision beads assay and normalized to untreated cells. Data are shown as mean ± SD of three independent experiments.

We first targeted *CD96* and *KLRC1* (encoding NKG2A), and quantitated the target protein expression by flow cytometry as a measure of KO efficiency. Parental NK-92 cells were 92.5 ± 0.9% double positive for CD96 and NKG2A with only 2.8 ± 3.4% of double negative cells. After editing, the cell population shifted to 47.9 ± 1.2% double negative, 37.3 ± 3.2% CD96 single negative and 4.8 ± 0.7% NKG2A single negative, leaving only 10 ± 1.8% of double positive cells (Figure 4A). The uneven sgRNA efficiencies likely reflected the difference between the ratios of CD96^−^ and NKG2A^−^ cells. Next, we targeted *CD96* and *NCR1* (encoding NKp46), and again observed high double KO efficiency. The parental cells were 5.3 ± 0.2% double negative, and became 81.3 ± 1.9% double negative after editing (Figure 4B). Finally, we combined all three sgRNAs to disrupt *CD96, KLRC1*, and *NCR1* at one time. The triple negative cells increased from 1.2 ± 0.7% in the parental cells to 50.9 ± 2.1% after editing (Figure 4C). In all three experiments, the cell viability was maintained at ~80% as seen in the RNP dosage experiment (Figure 2), suggesting that the cumulative dosage of multiple Cas9 RNPs were well-tolerated. These results demonstrate the efficiency of Cas9 RNP nucleofection for multiplex KO in NK-92.

The risk of chromosomal translocations is a concern when multiple DSBs are simultaneously induced by Cas9 and mis-ligated by NHEJ. After the triple KO experiment, we adopted a PCR-based assay to detect chromosomal translocations between the Cas9-edited sites (26). We used specific PCR primer sets to probe 12 possible translocation patterns between the *CD96, KLRC1*, and *NCR1* target sites on chromosome 3, 12, and 25, respectively (Figure S2A). In only the triple KO cells, we detected evidence of translocations in eight out of 15 patterns, but at much lower levels than the wild-type sequences (Figures S2B,C). Our results indicate that chromosomal translocation can occur between multiple Cas9-edited sites, giving rise to unexpected mutations that are easily missed by standard gene editing analyses.

### DNA Nucleofection Leads to Rapid Decline in Cell Viability

**Figure 5:**
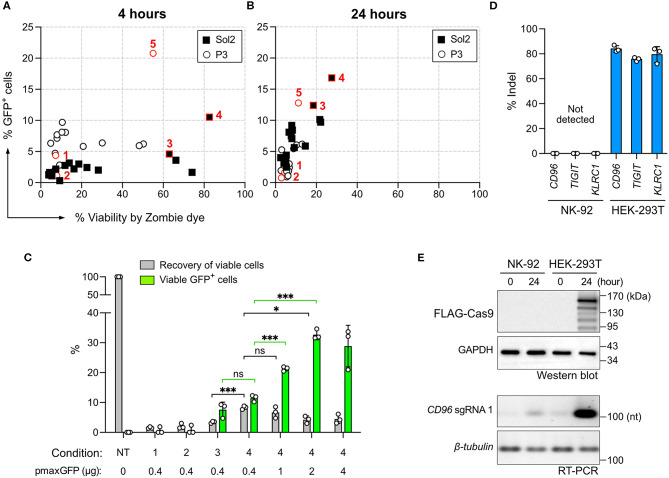
Screening of DNA nucleofection condition. The same 32 conditions used for Cas9 RNP nucleofection were tested for the nucleofection of the pmaxGFP plasmid. GFP expression and cell viability were determined by flow cytometry with Zombie dye staining at 4 h **(A)** and 24 h **(B)** post nucleofection. **(C)** A comparison of conditions 1–4 was performed using Precision beads assay. Increasing concentrations of the pmaxGFP plasmid were added to improve GFP expression in condition 4. Recovery of viable cells was normalized to the untreated condition (NT). GFP^+^ cells in the viable population were quantitated. **(D)** Plasmid-based CRISPR gene editing was performed to target *CD96, TIGIT*, and *KLRC1* genes in NK-92 and HEK-293T cells. No indel was detected in NK-92 by Sanger sequencing and ICE analysis. **(E)** Confirmation of Cas9 and sgRNA expression from the CRISPR plasmid by Western blotting and reverse-transcription PCR (RT-PCR). Cas9 protein carries a FLAG tag for detection. Cell samples were analyzed immediately (time = 0) and 24 h after nucleofection. GAPDH protein and β*-tubulin* mRNA serve as internal controls. Data are shown as mean ± SD of three independent experiments. Statistics by two-tailed Welch's unequal variances *t*-test; ns, not significant, *p ≤ 0.05 and ***p ≤ 0.001. The pulse codes, buffers and raw data are summarized in Table S1.

Cas9-mediated HDR requires the co-delivery of synthetic DNA repair templates to mediate DNA sequence exchange or gene insertion. Because the physicochemical and nucleofection properties of Cas9 RNP are distinct from those of DNA (31), we repeated the screening procedure for DNA nucleofection, aiming to find a suitable condition for co-delivery of Cas9 RNP and a DNA template. We tested the same 32 conditions as in Cas9 RNP nucleofection to deliver 0.4 μg of pmaxGFP plasmid, encoding turboGFP protein for detection. The expression of GFP was detected in as little as 4 h by flow cytometry (Figure 5A). Strikingly, cell viability declined rapidly in 24 h, with condition 4 being the most balanced condition with 17% GFP expression and 28% viability (Figure 5B).

DNA nucleofection appeared highly toxic to NK-92 cells. We performed the Precision beads assay to quantitate viable GFP^+^ cells in conditions 1–4. We did not pursue condition 5 because it was inefficient for RNP nucleofection (Figure 1C). Most of the cells died in condition 1 and 2 (Figure 5C). Condition 3 and 4 produced comparable percentages of viable GFP^+^ cells at 7.6 ± 2.5% and 11.5 ± 1%, respectively, but condition 4 yielded higher recovery of viable cells at 8.3 ± 0.6% (Figure 5C). Higher pmaxGFP concentrations at 1 and 2 μg helped increase viable GFP^+^ cells; however, 4 μg of pmaxGFP showed no further improvement (Figure 5C). Overall, DNA nucleofection led to significantly more cell death than by RNP nucleofection. Collectively, 1–2 μg of DNA in condition 4 is the best combination.

### Plasmid-Based CRISPR Gene Editing Is Ineffective

Plasmid-based CRISPR gene editing was reported to be ineffective in NK cells (19). We were curious whether this was also a problem for NK-92. We thus constructed three Cas9+sgRNA dual expressing plasmids, each encoding the best *CD96, TIGIT*, or *KLRC1* targeting sgRNA. We tested the three plasmids in NK-92 cells and also HEK293T cells as a control. We detected ~80% indel in HEK293T cells across all three loci, confirming robust editing of the three sgRNAs (Figure 5D). In stark contrast, there was no detectable indel in NK-92 cells in any target locus. We performed Western Blotting and reverse-transcription PCR to check Cas9 and sgRNA synthesis, respectively (Figure 5E). We detected both Cas9 and sgRNA in HEK293T cells, but no Cas9 and only a hint of sgRNA in NK-92 cells, reflecting the lack of gene editing. These results emphasize the superiority of Cas9 RNP gene editing in NK-92 over the plasmid approach.

### Cas9-Mediated HDR Allows Non-viral Gene KI

**Figure 6:**
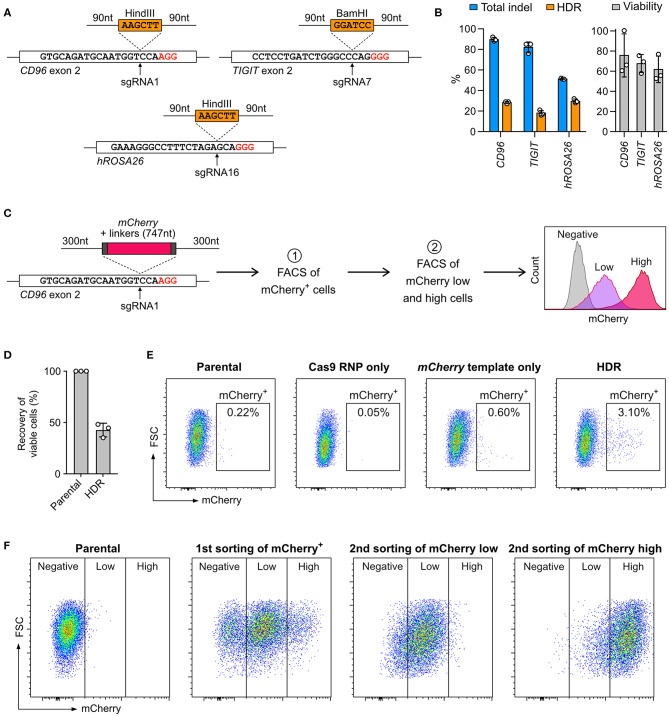
Knock-in (KI) by Cas9 RNP-mediated homology-directed repair (HDR). **(A)** Co-nucleofection of DNA ultramer, as HDR templates, allows the insertion of either BamHI or HindIII restriction sites at the indicated sgRNA target sites. The upstream and downstream homology arms are 90-nt long. PAM sequences are marked in red. **(B)** Total indels and HDR frequencies were determined by Sanger sequencing and ICE analysis. The normalized cell viability shows that single-strand DNA is less toxic than pmaxGFP to NK-92. Data are shown as mean ± SD of three independent experiments. **(C)** A double-stranded PCR template was used to facilitate the insertion of a promoter-less *mCherry* reporter gene in-frame into the *CD96* exon 2 at the sgRNA1 target site. The homology arms are 300-nt long. The expression of mCherry, as driven by the endogenous *CD96* promoter, was analyzed by flow cytometry. Two rounds of fluorescence-activated cell sorting (FACS) were conducted to first enrich mCherry^+^ cells and then further isolate mCherry low-expressing and mCherry high-expressing cells. **(D)** Viability assay reveals moderate level of toxicity of double-stranded PCR templates. **(E)** Flow cytometry plots show the percentages of mCherry^+^ cells at 72 h after nucleofection. **(F)** Distributions of mCherry low-expressing and high-expressing cells are shown after each round of cell sorting.

Cas9-mediated HDR allows precise sequence modifications and targeted gene insertion; however, this approach is not effective in all cell types. To test whether NK-92 cells are capable of HDR, we inserted restriction sites into the *CD96* and *TIGIT* loci and *hROSA26* genome safe harbor (Figure 6A). The restriction sites were encoded on synthetic DNA oligonucleotides (DNA ultramer) and flanked by 90-nt homology arms. We nucleofected NK-92 cells with 100 pmol (equivalent to 0.06 μg due to low molecular weight) of DNA ultramer using condition 4. We detected 20–30% HDR efficiencies across the three target loci by ICE analysis (Figure 6B). Although the percent total indel at *hROSA26* was lower than those at *CD96* and *TIGIT*, the HDR efficiency at *hROSA26* was comparable to the others. Cell viability was maintained at 60–80% at all three loci, because the concentration of DNA ultramer was much lower than that of pmaxGFP. Our results demonstrate that NK-92 can utilize DNA ultramer as HDR templates for short sequence modifications, opening the possibility to introduce point mutations and protein fusion tags.

Next, we integrated the *mCherry* reporter gene in-frame into *CD96* exon 2, by adopting a similar strategy from Roth et al. (32) The *mCherry* sequence was encoded on a PCR-synthesized double-stranded DNA (dsDNA) and flanked by 300-nt homology arms (Figure 6C; full DNA sequence in Table S4). The expression of mCherry was driven by the *CD96* endogenous promoter and quantitated by flow cytometry. After editing, the viability was 42.7 ± 6.7%, indicating that the 1,695-nt *mCherry* template was moderately toxic as compared to 186-nt DNA ultramer and 3,487-nt pmaxGFP (Figure 6D). About 3% of the edited cells were mCherry^+^. The *mCherry* template-only control had 0.6%, likely due to non-specific genomic integration (Figure 6E). The KI efficiency of *mCherry* was significantly lower than that of restriction sites, revealing the challenge of introducing gene-sized modifications in NK-92 cells by Cas9-mediated HDR.

Being a lymphoma cell-type, NK-92 cells are capable of clonal expansion. We took advantage of this and isolated the mCherry^+^ cells by FACS for expansion. To ensure accurate insertion, we validated the genomic junctions flanking the *mCherry* insert by Sanger sequencing (Figure S3). In the second round of FACS, the mCherry^+^ population was further separated into low-expressing and high-expression groups (Figure 6F). The enriched cells stably retained their mCherry expression levels after multiple passages and cryopreservation. Our results show that Cas9-mediated HDR coupled with FACS enrichment is a useful strategy to enable precise genome editing and overcome low HDR efficiency. Unfortunately, we could not isolate single clones because NK-92 did not grow from a single cell. Overcoming this technical hurdle is necessary to create genetically defined NK-92 clones for therapeutic applications.

### Cas9-Mediated Promoter Insertion Reactivates Silenced Genes

**Figure 7:**
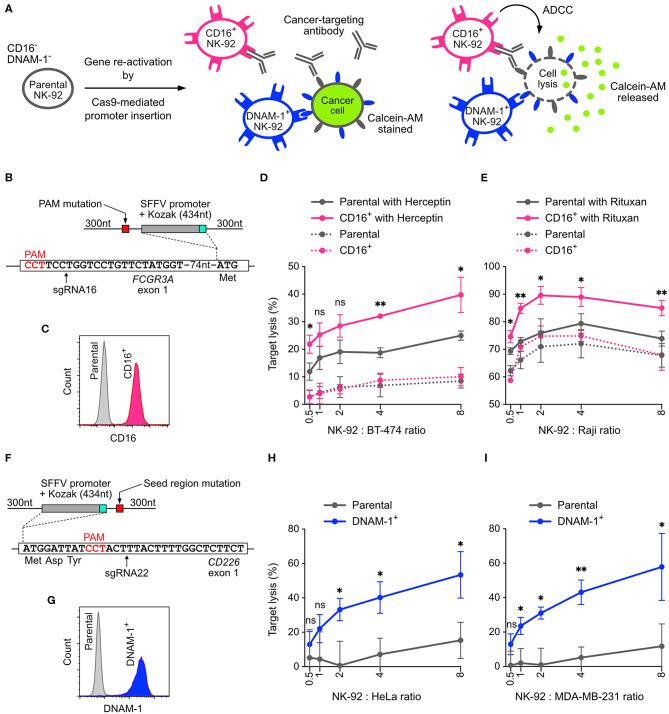
Reactivation of CD16 and DNAM-1 by Cas9-mediated promoter insertion. **(A)** Parental NK-92 cells do not express CD16 and DNAM-1. Reactivation of CD16 enables NK-92 cells to execute antibody-dependent cellular toxicity (ADCC) in combination with a cancer-targeting antibody. Reactivation of DNAM-1 allows NK-92 to recognize CD155 or CD112 ligand on cancer cells and initiates cytotoxicity. In the cytotoxicity assay, lysis of the cancer cells is measured by the release of Calcein-AM. **(B)** A double-stranded HDR template facilitates the insertion of SFFV and Kozak sequences immediately upstream of the *FCGR3A* start codon. The sgRNA16-guided Cas9 cleavage site is marked by arrow. The amino acids indicate the immediate N-terminal peptide sequence. The PAM sequence of the sgRNA16 target site was mutated in the HDR template to avoid targeting by Cas9. **(C)** Flow cytometry plot shows enriched CD16^+^ cells. **(D)** Lysis of BT-474 cells in Herceptin-mediated ADCC in CD16^+^ NK-92 cells vs. parental cells. **(E)** Lysis of Raji cells in Rituxan-mediated ADCC. **(F)** In a similar setup, SFFV and Kozak sequences were inserted before the *CD226* start codon, at the cleavage site defined by sgRNA22 (arrow). The seed region of sgRNA22 target site was modified by silent mutations in the HDR template to avoid targeting by Cas9. **(G)** Flow cytometry plot shows enriched DNAM-1^+^ cells. Lysis of HeLa **(H)** and MDA-MB-231 **(I)** cancer cells by DNAM-1^+^ NK-92. Data are shown as mean ± SD of three independent experiments. Statistics by two-tailed Welch's unequal variances *t*-test; ns, not significant, *p ≤ 0.05 and **p ≤ 0.01. DNA sequences of HDR templates are in Table S4.

NK-92 cells is less potent than primary NK cells because some cytotoxicity-related genes are silenced. The common approach to restore these gene activities is to insert the cDNA of the silenced genes into NK-92 genome using viral transduction. However, the genomic insertion of viral vectors occurs at random sites, producing a heterogeneous population of cells with mixed genotypes and potentially uneven activities. To demonstrate that non-viral, site-specific genome editing is a better approach, we designed Cas9-mediated HDR to reactivate the endogenous genes by replacing the silenced promoter with a spleen focus-forming virus (SFFV) promoter (33). We targeted the *FCGR3A* and *CD226* genes, which encode CD16 and DNAM-1, respectively (Figure 7A). CD16 is a surface receptor that binds to the Fc region of IgG antibodies and is essential for antibody-dependent cellular cytotoxicity (ADCC) in NK cells (34). DNAM-1 binds to the tumor-associated antigens CD155 and CD112 and synergizes cytotoxicity with other activating receptors (35).

Parental NK-92 cells do not express CD16 and therefore cannot mediate ADCC. We programmed Cas9 RNP to target near the start codon of *FCGR3A*, in order to insert the SFFV promoter and Kozak sequence immediately upstream of the start codon (Figure 7B). The sgRNA selection was limited in this region, because most of the sgRNA candidates had low off-target scores and low editing efficiencies, except sgRNA16 (Figure 3). We anticipated low HDR efficiency because the cleavage site of sgRNA16 was 91-nt away from the intended insertion site. After HDR, we obtained 1.2% of CD16^+^ cells. We were able to isolate the CD16^+^ cells by FACS and expanded to 97.4% purity (Figure S4A). The expression of CD16 was stable after clonal expansion and cell passage (Figure S4 and Figure 7C). We also verified the genomic junctions flanking the SFFV cassette by Sanger sequencing (Figure S4).

CD16^+^ NK-92 was then assayed *in vitro* for ADCC against cancer cell lines. In the presence of Herceptin (anti-HER2 antibody), CD16^+^ NK-92 showed 2-fold enhancement in cytotoxicity against BT474 (HER2^+^ ductal carcinoma) compared to the parental cells at various NK-92-to-cancer ratios (Figure 7D). The combination of Herceptin and parental NK-92 also led to some increase in cancer lysis, likely due to the direct action of Herceptin against BT474. Similar levels of enhancement were also observed with Rituxan (anti-CD20 antibody) against Raji, a CD20^+^ B cell lymphoma (Figure 7E). The results demonstrate that the cytotoxicity of NK-92 benefits significantly from reactivation of CD16 and ADCC.

Using the same HDR strategy, we also reactivated DNAM-1 expression by SFFV insertion, validated the genomic junctions, FACS enriched and clonal expanded the DNAM-1^+^ cells (Figures 7F, G and Figure S5). We then assayed DNAM-1^+^ NK-92 against HeLa and MDA-MB-231 cancer cell lines, which express high levels of CD155 and are challenging to kill by parental NK-92 cells. The expression of DNAM-1 activating receptor significantly boosted the cytotoxicity by 4-fold against both cancer cells across various NK-92-to-target ratios (Figures 7H,I). In summary, our results demonstrate that both CD16 and DNAM-1 enhance the cytotoxicity of NK-92 cells against cancer cell lines *in vitro*. The promoter-insertion strategy proves the possibility to site-specifically reactivate any endogenous genes by Cas9-mediated HDR, creating improved versions of NK-92 cells with well-defined genotypes.

## Discussion

NK-92 cells are a clinically valuable cell line that can help overcome the shortage of primary NK cells for adoptive immunotherapy. A robust and site-specific genome-editing tool is vital for functional study and therapeutic engineering of NK-92 cells. We describe a CRISPR genome editing platform for these cells based on the nucleofection of Cas9 RNP. This approach offers fast action, high efficiency, multiplex capability, low toxicity, and does not involve exogenous genetic materials in the forms of plasmid or viral DNA. Co-nucleofection of the DNA repair template allows synchronization of Cas9-mediated DNA cleavage and HDR to integrate exogenous DNA sequences into the targeted genomic loci. This is a popular approach for genome editing in a variety of human cells, and now a robust platform is also established for NK-92 cells.

Several interesting observations were made during the optimization of Cas9 RNP and DNA nucleofection. First, parallel comparison reveals that primary NK and NK-92 cells require different a nucleofection protocol. Overall, Sol2 is better than P3 for NK-92. Sol2 is more affordable, composed of common laboratory chemicals, and can potentially be fine-tuned to further improve cell viability and Cas9 RNP and DNA delivery. It is also possible to scale up the Lonza nucleofection reaction, using larger nucleofection cuvettes, to increase NK-92 cell production.

Second, DNA toxicity is a major hurdle in the introduction of exogenous DNA sequences. The pmaxGFP experiment reveals that, while it is possible to increase DNA delivery and transgene expression by using higher DNA dosages, NK-92 cells die rapidly in response to DNA nucleofection. Toxicity levels appear to correlate with DNA concentration and length, judging from the HDR experiments using a DNA ultramer, a mCherry dsDNA PCR template and a pmaxGFP plasmid. While the toxicity of DNA ultramer is the lowest, the length limitation (200 nt) of the synthetic oligonucleotides excludes the possibility of incorporating a promoter sequence or a gene. We have not compared the cell viability or HDR efficiency using single-stranded vs. double-stranded template of the same length. It would be interesting to convert a long dsDNA template into a single-stranded form, using the commercially available kits, to see if the toxicity problem is alleviated.

We are currently investigating intracellular DNA immunity in primary NK and NK-92 cells to understand the mechanism of DNA toxicity. Because NK cells are a pivotal component of innate immunity against viral infections, they likely possess a comprehensive set of intracellular sensing and defense mechanisms against DNA of foreign origins. The activation of intracellular DNA immunity, for example the cGAS-STING pathway, is known to induce rapid inflammatory responses, pyroptosis and apoptosis (36). Similar observations were made previously in primary lymphocytes, where the nucleofection of exogenous DNA induced inflammatory responses and apoptosis (37, 38). Whether or not intracellular DNA immunity is responsible for DNA-induced NK-92 cell death awaits experimental confirmation. Elucidation of DNA toxicity could offer valuable insight into the design of HDR templates that can evade immune detection in NK cells.

In contrast to DNA, nucleofection of high dosages of Cas9 RNP (up to 120 pmol per 4 × 10^5^ cells) seems well-tolerated in NK-92 cells. No significant increase in KO efficiency was observed beyond 40 pmol at the *CD96* and *NCR1* loci, suggesting that higher RNP dosage is unnecessary for single KO. On the other hand, targeting essential genes is expected to have a detrimental effect on cell viability, especially when high KO efficiency obliterates completely two alleles. We suspect this is the case in the *KLRK1* KO. When the essential NKG2D receptor, encoded by *KLRK1*, is eliminated, NK-92 cells can no longer survive. However, we cannot completely rule out the possibility that off-target effects at high RNP dosage contribute to the death of *KLRK1* KO cells. Our amplicon-based NGS did not detect any sequence deviation at the two predicted off-target sites, but a more extensive investigation is necessary to confirm the integrity of genome.

Cas9 RNP nucleofection is a robust approach to perform double and triple KO in NK-92. When coupled with FACS, KO mutants can be isolated, expanded and assayed for the loss of gene functions. The molar ratio of the RNP pool can be adjusted without difficulty *in vitro* to compensate for low KO efficiency of certain sgRNAs. Such adjustment cannot be made in plasmid-based editing because neither nucleofection nor chemical transfection can guarantee precise expression levels of the cargo genes. In fact, plasmid-based editing was completely ineffective in NK-92. The lack of Cas9 and sgRNA expression was the main reason that our three independent KO attempts failed to produce detectable indels.

Although multiplexed gene KO by Cas9 RNP is highly efficient, we have shown that chromosomal translocation can happen in NK-92 between Cas9 edited sites on different chromosomes due to mis-ligation by NHEJ pathway. Chromosomal translocations can produce unexpected mutations and phenotypes that are not easily detected by the standard amplicon-based gene editing analyses and functional assays, respectively. The ability to isolate clonal NK-92 cells is therefore necessary to obtain a homogeneous genotype of the edited cells and verify whole genome integrity. This process is not yet possible for NK-92 because single cell expansion remains a challenge.

Cas9-mediated HDR was successful in NK-92 cells, although the HDR efficiency decreased as the length of the DNA insert and HDR template increased. Insertion of restriction sites and the *mCherry* gene occurred at ~20 and 3%, respectively, indicating that NK-92 cells are capable of HDR using both ssDNA ultramer and dsDNA PCR templates. Moreover, Cas9-mediated promoter insertion effectively reactivated the endogenous *FCGR3A* and *CD226* enhanced NK-92 cytotoxicity. This strategy demonstrates the feasibility of site-specifically reactivating endogenous genes by Cas9-mediated HDR, and offers an attractive alternative to viral transduction, where viral integration may perturb genome integrity and produce cell-to-cell variation. The length of the SFFV promoter is also shorter than a full transgene and can be encoded in a shorter HDR template. We anticipate that by optimizing the homology arms, switching to ssDNA template, or incorporating NHEJ and cell cycle regulators, HDR efficiency may be further improved.

In summary, we describe a highly efficient CRISPR platform for genome engineering of NK-92 cells. The nucleofection protocols, the multiplex KO and KI strategies, and functional analyses are robust and readily adaptable for the development of NK-92 therapeutics. However, DNA toxicity still remains a major obstacle that interferes with the HDR process and the recovery of viable KI cells. Overcoming this toxicity would improve HDR and allow engineering more novel functions into NK-92 cells. Such functions are expected to match or exceed the primary level of competency.

## Data Availability Statement

The datasets generated for this study can be found in the NCBI Sequence Read Archive (PRJNA608597).

## Author Contributions

R-SH, H-AS, M-CL, and SL conceived and designed this study. R-SH, H-AS, and M-CL performed the experiments. R-SH and Y-JC analyzed the NGS results. R-SH and SL wrote the manuscript.

## Conflict of Interest

The authors declare that the research was conducted in the absence of any commercial or financial relationships that could be construed as a potential conflict of interest.

## Supplementary Material

Figure S1 Cell viability assay by precision cell count beads. **(A)** Workflow of precision beads assay. **(B)** Flow cytometry data of the untreated control and nucleofected cells.

Figure S2 Detection of chromosomal translocation in the CD96-KLRC1-NCR1 triple KO cells by a PCR-based assay. **(A)** Schematic representation of the three target loci showing the PCR primers, Cas9 RNP cleavage positions, and the expected size of PCR fragments. **(B)** Nine possible combinations of forward and reverse primers were used to detect different arrangements of chromosomal translocation. The presence of DNA bands indicates evidence of chromosomal translocation in the triple KO cells. **(C)** Six possible combinations of forward primers were used to detect chromosomal inversion. All four DNA gels contained the same amount of DNA ladder for parallel comparison.

Figure S3 Validation of *mCherry* knock-in by Sanger sequencing using PCR primer sets that were specific for genomic DNA, but not the HDR template. The genomic junctions upstream and downstream from the insert were sequenced to confirm precise HDR.

Figure S4 **(A)** Flow cytometry analysis of CD16 expression after HDR and FACS enrichment. **(B)** The insertion of SFFV promoter was validated by Sanger sequencing using PCR primer sets that were specific for genomic DNA, but not the HDR template. The PAM sequence of sgRNA16 target site was mutated in the HDR template to avoid targeting by Cas9.

Figure S5 **(A)** Flow cytometry analysis of DNAM-1 expression after HDR and FACS enrichment. **(B)** The insertion of SFFV promoter was validated by Sanger sequencing using PCR primer sets that were specific for genomic DNA, but not the HDR template. The seed region of sgRNA22 target site was modified to silent mutations in the HDR template to avoid targeting by Cas9.

Table S1 Raw data of Cas9 RNP and pmaxGFP nucleofection screening.

Table S2 sgRNA list and gene editing efficiencies.

Table S3 PCR primers for genomic DNA amplification, plasmid construction and NGS.

Table S4 Full DNA sequences of the HDR templates.

## Abbreviations

ADCC: antibody-dependent cellular cytotoxicity
CRISPR: clustered regularly interspaced short palindromic repeats
FACS: fluorescence-activated cell sorting
dsDNA: double-stranded DNA
HDR: homology-directed repair
indel: insertion-deletion
IVT: *in vitro* transcription
KI: knock-in
KO: knockout
NGS: next generation sequencing
NHEJ: non-homologous end joining
NK: natural killer
RNP: ribonucleoprotein
NT: untreated
SFFV: spleen focus-forming virus
sgRNA: single guide RNA
Sol2: solution2+mannitol
ssDNA: single-stranded DNA
7-AAD: 7-aminoactinomycin D.
