# Supplementary figures and images for: Enhanced NK-92 Cytotoxicity by CRISPR Genome Engineering Using Cas9 Ribonucleoproteins
Source: Front Immunol. 2020 May 22;11:1008. doi: 10.3389/fimmu.2020.01008 (PMC7256201; doi:10.3389/fimmu.2020.01008)

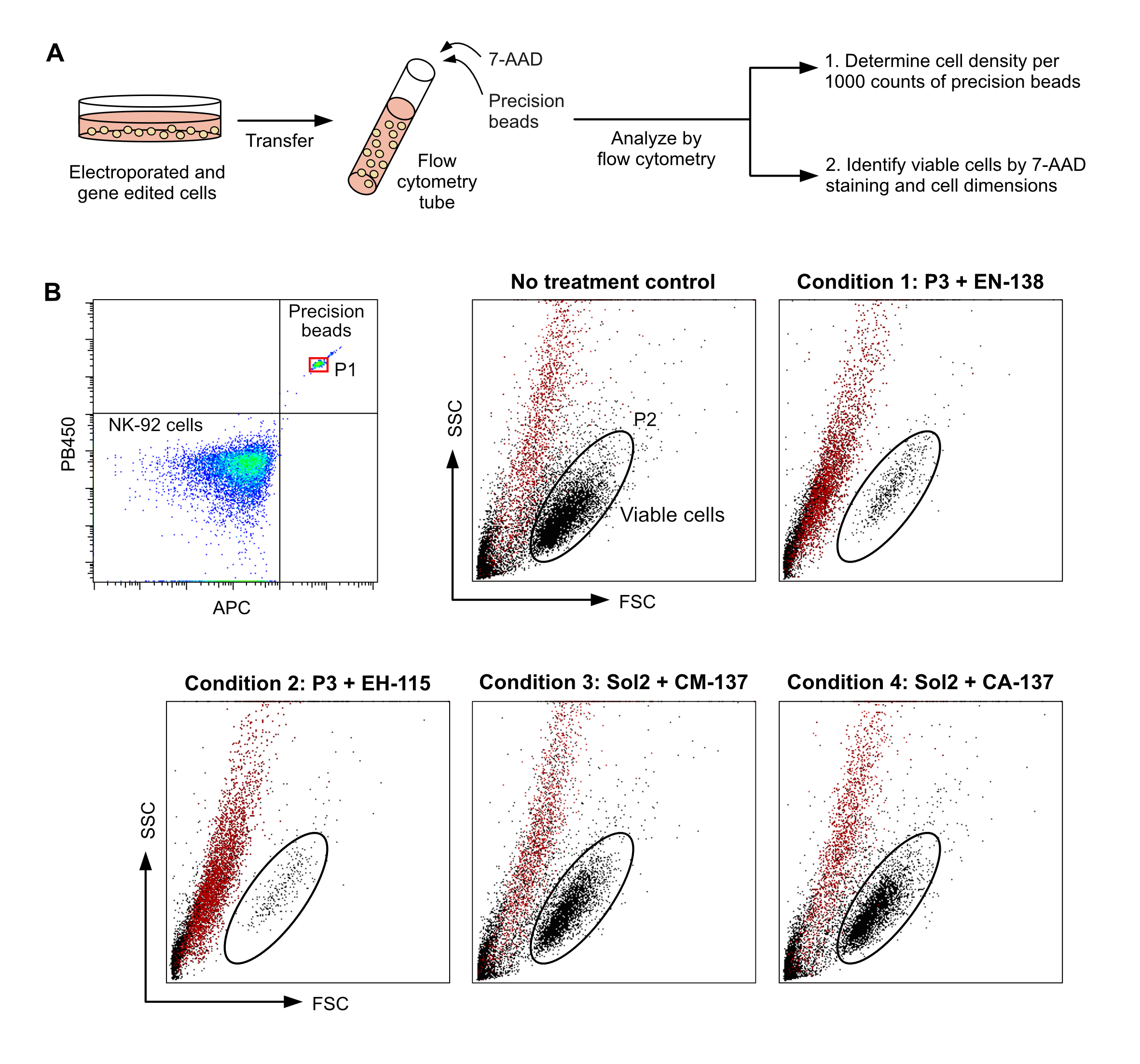

Supplement: Figure S1 — Cell viability assay by precision cell count beads. (A) Workflow of precision beads assay. (B) Flow cytometry data of the untreated control and nucleofected cells. [file Image_1.tiff]

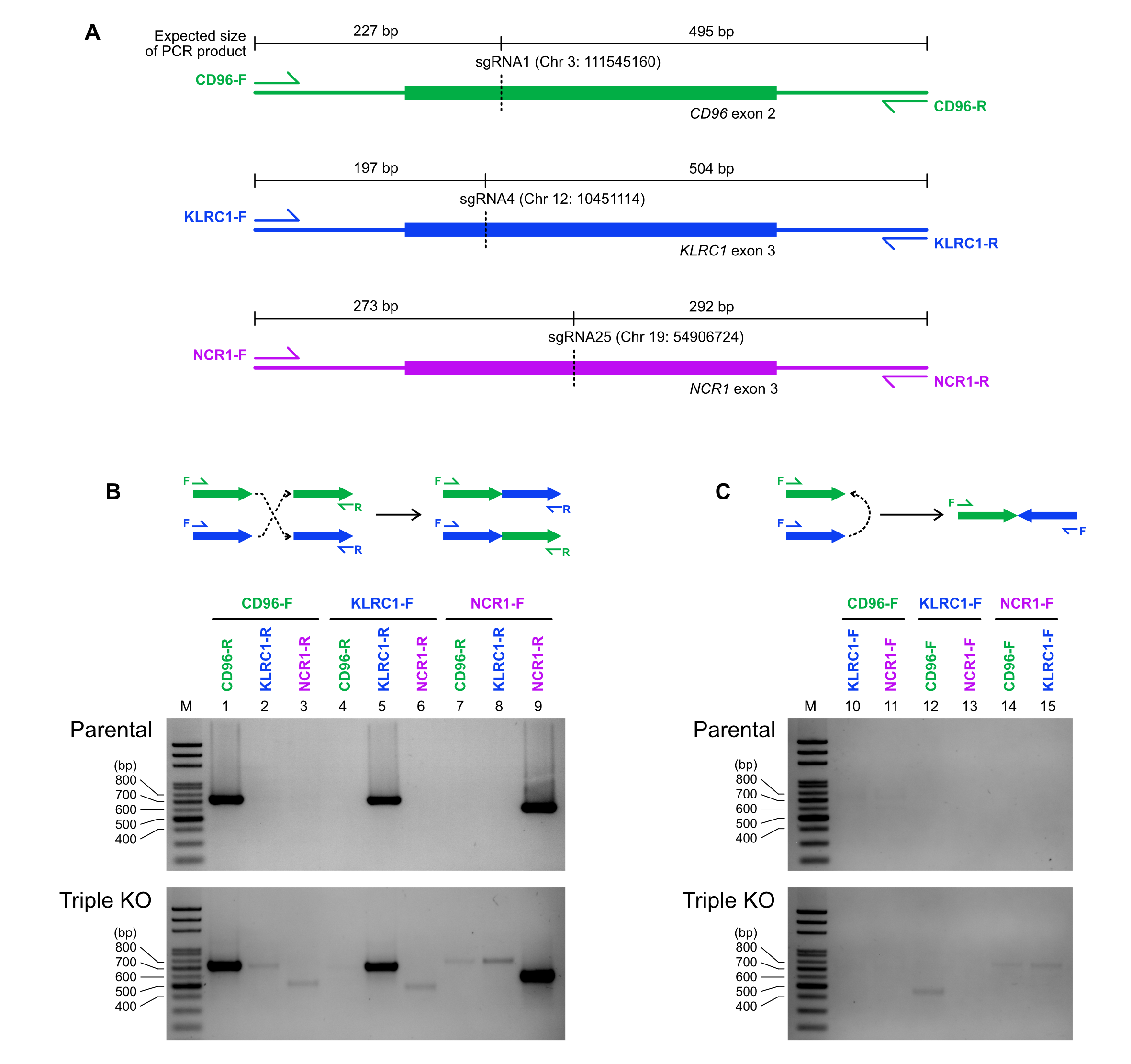

Supplement: Figure S2 — Detection of chromosomal translocation in the CD96-KLRC1-NCR1 triple KO cells by a PCR-based assay. (A) Schematic representation of the three target loci showing the PCR primers, Cas9 RNP cleavage positions, and the expected size of PCR fragments. (B) Nine possible combinations of forward and reverse primers were used to detect different arrangements of chromosomal translocation. The presence of DNA bands indicates evidence of chromosomal translocation in the triple KO cells. (C) Six possible combinations of forward primers were used to detect chromosomal inversion. All four DNA gels contained the same amount of DNA ladder for parallel comparison. [file Image_2.tiff]

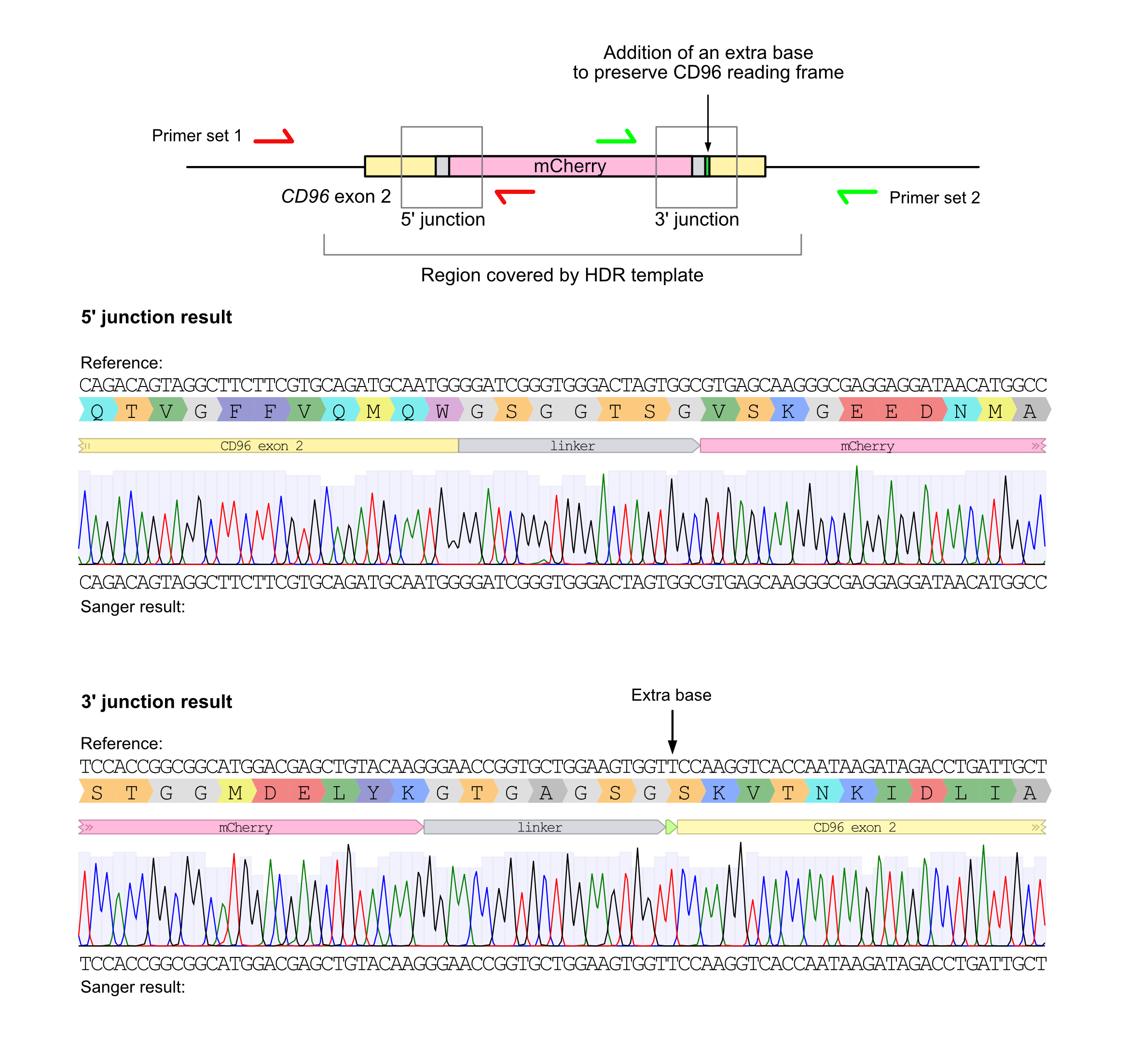

Supplement: Figure S3 — Validation of mCherry knock-in by Sanger sequencing using PCR primer sets that were specific for genomic DNA, but not the HDR template. The genomic junctions upstream and downstream from the insert were sequenced to confirm precise HDR. [file Image_3.tiff]

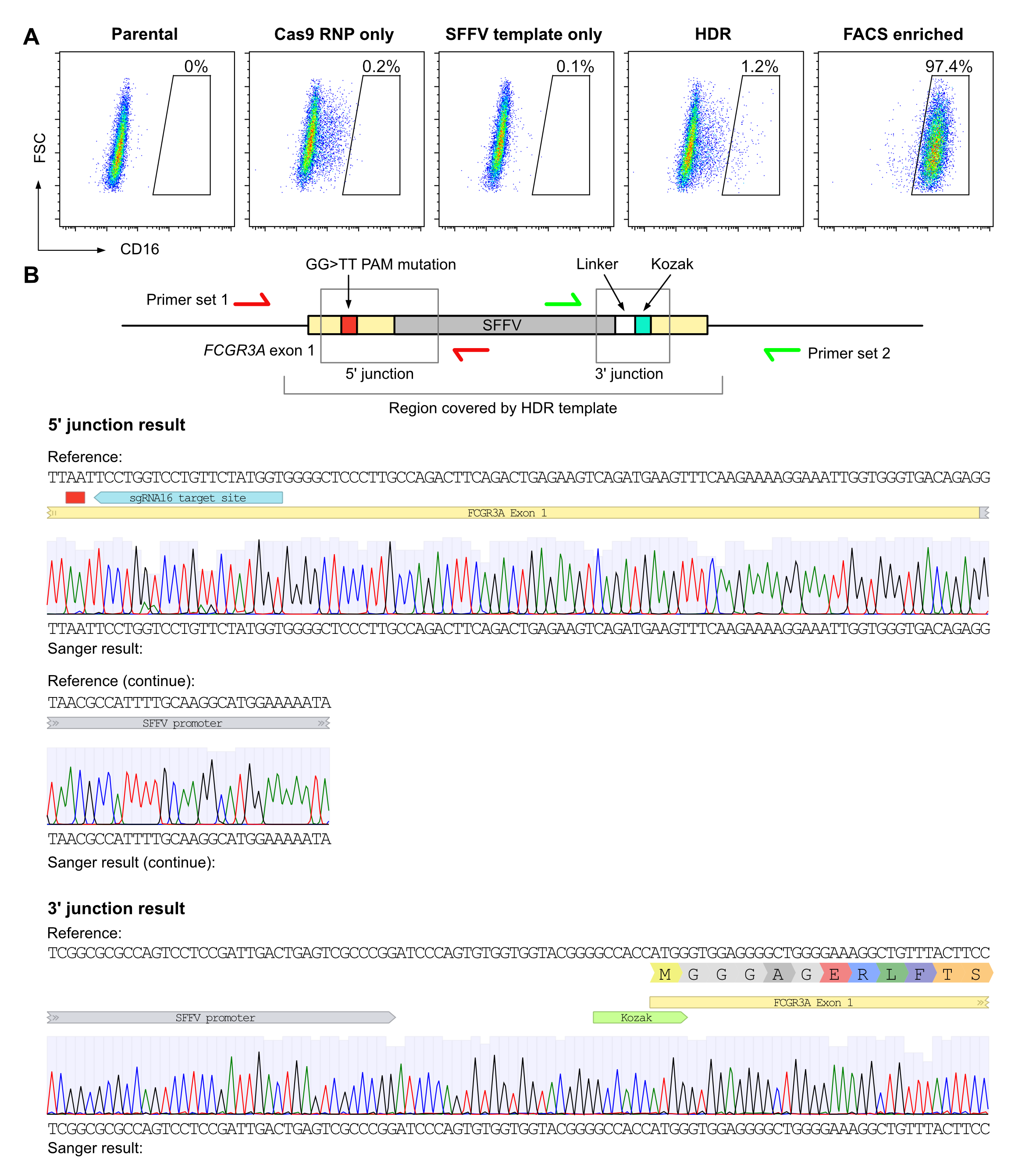

Supplement: Figure S4 — (A) Flow cytometry analysis of CD16 expression after HDR and FACS enrichment. (B) The insertion of SFFV promoter was validated by Sanger sequencing using PCR primer sets that were specific for genomic DNA, but not the HDR template. The PAM sequence of sgRNA16 target site was mutated in the HDR template to avoid targeting by Cas9. [file Image_4.tiff]

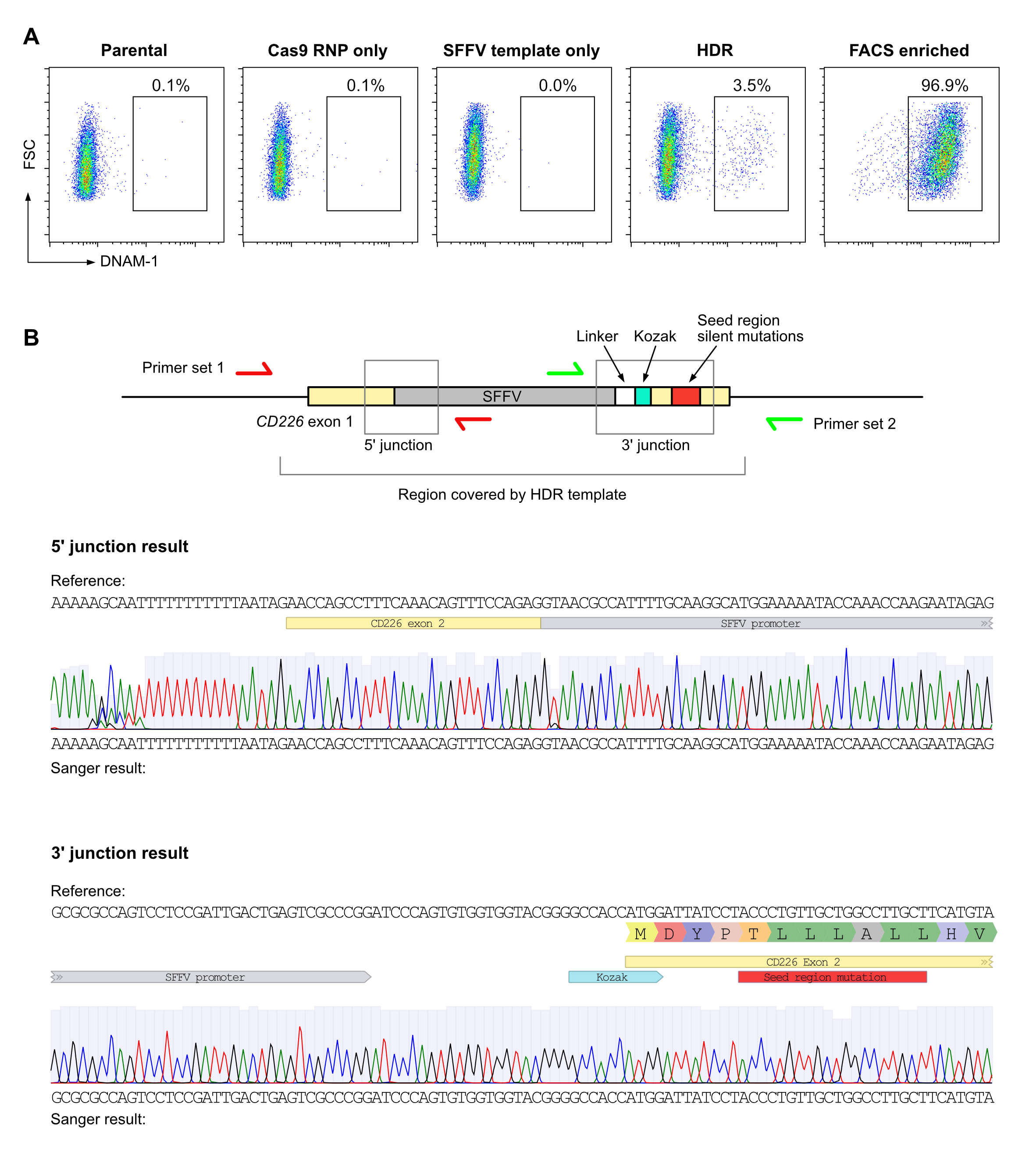

Supplement: Figure S5 — (A) Flow cytometry analysis of DNAM-1 expression after HDR and FACS enrichment. (B) The insertion of SFFV promoter was validated by Sanger sequencing using PCR primer sets that were specific for genomic DNA, but not the HDR template. The seed region of sgRNA22 target site was modified to silent mutations in the HDR template to avoid targeting by Cas9. [file Image_5.tiff]
