# Supplementary material for: Enhanced NK-92 Cytotoxicity by CRISPR Genome Engineering Using Cas9 Ribonucleoproteins
Source: Front Immunol. 2020 May 22;11:1008. doi: 10.3389/fimmu.2020.01008 (PMC7256201; doi:10.3389/fimmu.2020.01008)
Supplement: Table S1 — Raw data of Cas9 RNP and pmaxGFP nucleofection screening. [file Table_1.DOCX]

| Buffer and  pulse code | | CD96 RNP at 72h | | pmaxGFP (0.4 μg) at 4h | | pmaxGFP (0.4 μg) at 24h | |
| --- | --- | --- | --- | --- | --- | --- | --- |
|  |  | % Viability | % CD96 negative | % Viability | % GFP+ | % Viability | % GFP+ |
| Sol2 | CA-137 | 96.45 | 89.85 | 82.61 | 10.53 | 27.52 | 16.83 |
|  | CM-138 | 94.94 | 60.28 | 66.20 | 3.61 | 22.10 | 9.72 |
|  | CM-137 | 93.74 | 75.84 | 62.96 | 4.61 | 18.53 | 12.41 |
|  | CM-150 | 96.45 | 38.84 | 74.14 | 1.65 | 21.69 | 10.12 |
|  | DN-100 | 52.60 | 26.38 | 11.86 | 2.78 | 7.73 | 7.52 |
|  | DS-138 | 80.91 | 23.90 | 16.25 | 2.22 | 8.04 | 7.08 |
|  | DS-137 | 62.40 | 31.43 | 13.94 | 3.16 | 6.61 | 8.10 |
|  | DS-130 | 81.73 | 16.53 | 22.65 | 2.43 | 7.99 | 9.19 |
|  | DS-150 | 87.50 | 26.45 | 18.66 | 3.00 | 8.27 | 8.42 |
|  | DS-120 | 94.61 | 18.48 | 28.23 | 2.02 | 14.59 | 5.87 |
|  | EH-100 | 27.43 | 19.08 | 3.89 | 1.29 | 5.36 | 2.49 |
|  | EO-100 | 46.69 | 18.36 | 7.35 | 2.18 | 9.21 | 5.62 |
|  | EN-138 | 45.22 | 17.12 | 5.44 | 1.10 | 5.27 | 4.40 |
|  | EN-150 | 43.63 | 16.00 | 4.27 | 1.64 | 5.05 | 3.36 |
|  | EW-113 | 47.18 | 15.06 | 4.04 | 1.24 | 2.58 | 4.01 |
|  | EH-115 | 11.25 | 8.66 | 9.25 | 0.32 | 4.09 | 3.09 |
| P3 | CA-137 | 65.09 | 41.22 | 55.05 | 20.80 | 11.17 | 12.79 |
|  | CM-138 | 37.86 | 21.22 | 50.33 | 6.22 | 10.30 | 6.03 |
|  | CM-137 | 40.96 | 16.53 | 30.12 | 6.37 | 8.87 | 5.46 |
|  | CM-150 | 41.96 | 18.21 | 48.22 | 5.95 | 13.07 | 6.19 |
|  | DN-100 | 31.54 | 8.40 | 7.99 | 7.13 | 5.92 | 1.88 |
|  | DS-138 | 41.28 | 14.22 | 7.92 | 7.70 | 6.76 | 2.42 |
|  | DS-137 | 23.83 | 13.68 | 7.01 | 4.71 | 2.70 | 1.93 |
|  | DS-130 | 23.92 | 12.67 | 12.12 | 8.09 | 5.22 | 2.83 |
|  | DS-150 | 28.19 | 13.87 | 10.54 | 9.68 | 3.06 | 5.25 |
|  | DS-120 | 44.72 | 18.16 | 20.79 | 5.82 | 6.68 | 3.01 |
|  | EH-100 | 36.47 | 18.10 | 7.08 | 2.12 | 5.57 | 0.94 |
|  | EO-100 | 45.49 | 15.59 | 10.34 | 8.12 | 6.41 | 1.85 |
|  | EN-138 | 24.26 | 19.46 | 7.30 | 4.38 | 4.53 | 1.64 |
|  | EN-150 | 29.35 | 31.86 | 4.89 | 6.34 | 5.44 | 1.79 |
|  | EW-113 | 21.71 | 23.54 | 9.22 | 2.82 | 6.15 | 1.10 |
|  | EH-115 | 11.88 | 8.80 | 7.90 | 1.14 | 2.89 | 0.76 |

**Screening of Cas9 RNP and DNA nucleofection in Fig 1C, 4A and 4B.**
