# Supplementary material for: Enhanced NK-92 Cytotoxicity by CRISPR Genome Engineering Using Cas9 Ribonucleoproteins
Source: Front Immunol. 2020 May 22;11:1008. doi: 10.3389/fimmu.2020.01008 (PMC7256201; doi:10.3389/fimmu.2020.01008)
Supplement: Table S3 — PCR primers for genomic DNA amplification, plasmid construction and NGS. [file Table_3.DOCX]

| Primers for genomic amplification for ICE analysis and chromosomal translocation assay | | | |
| --- | --- | --- | --- |
| Locus | Exon | Amplicon | Primer (5’-3’) |
| CD96 | 2 | 722 bp | F: GGGAGAAATTTCCTCAGTTGCTCCCCTCAC  R: TCAATGGCCTCTGTGATGTGCTCCTTCACA |
| KLRC1 | 3 | 701 | F: CGTGTTTAGGCTTGAAGAAAATCAGAGAAAG  R: TTCACAGACAAGAGAACCTGAATGCACAGG |
| TIGIT | 2 | 688 bp | F: GCTCACCCTATGCAGTTTGTGTATTTGTTG  R: ATCAGTCTCTAGTGGTAGAACCTCTGGAAT |
| SIGLEC7 | 1 | 737 bp | F: GCCTCTTCTAAGTCTTGAGCCCGCAGTTCC  R: GTCAGAGGCAGGATGAGGATGTCAGGCCTGTG |
| NCR1 | 3 | 565 bp | F: CAGCAGCTGGGTGGAGCCTAAGGTTG  R: CTGACTGAGGACACATCTGCTCCTAGGACG |
| KLRK1 | 4 | 765 bp | F: GACTGGGGAACTTGTAGAGAAGTATAGAAGG  R: GATGATGTCAAACCTAAGTAGCCTCTAC |
| CD226 | 2 | 779 bp | F: GAGAGAAGAAACTCAAATCTGCCCATCCTC  R: GTCATGTTATTGGAAGCCATCGTTGAATTC |
| FCGR3A | 1 | 665 bp | F: GACAAGTTCTGAGGTGAAATGAAGGAAGCC  R: CATTCTCCTCATTTCTAGCCCCATCTTGGC |

| Primers for NGS analysis | | |
| --- | --- | --- |
| Locus | Amplicon | Primer (5’-3’) |
| CD96 on-target | 248 bp | F: GTTTATGCTACACTTGGCTCTG  R: CTGTGAAAGTCACAAGTGACTC |
| CD96 off-target-1 | 255 bp | F: GTTAGTACAGTAATTCAATGCAAC  R: CCCTAGCACAAAGTGGTTGTTC |
| CD96 off-target-2 | 284 bp | F: CCATTTTCCATCTCTAATGATGCCAG  R: GAGGCATTGCCTGGTCAGTTAC |
| KLRK1 on-target | 251 bp | F: GATACCCAAACCTTTTATGC  R: GACAATGTTGCAATCTACTTC |
| KLRK1 off-target-1 | 283 bp | F: CACAATTTCCTACATAGGAG  R: GATGTCCAGGTTTTCAAATC |
| KLRK1 off-target-2 | 284 bp | F: TCTGGCAAAATTTTGGCTAC  R: GATGCAACTCCTTTTTCGCC |

| Primers for HDR template production | | |
| --- | --- | --- |
| HDR template | Amplicon | Primer (5’-3’) |
| CD96-mCherry | 1369 bp | F: GCTTCTCTACGTAAATGCCTCCTC  R: GTACATTTGTTGAATGAGAGAGAGCTG |
| SFFV-FCGR3A | 1070 bp | F: GAAATGAAGGAAGCCCTCAGAG  R: CTAGCCCCATCTTGGCTTGTCCTAG |
| SFFV-CD226 | 1054 bp | F: GAGTTAGGCACAGAGAATGCATGC  R: GTTAGCCTTGATGATGGAAACACC |

| Primers for HDR validation | | |
| --- | --- | --- |
| Junction | Amplicon | Primer (5’-3’) |
| CD96-mCherry  5’ junction | 769 bp | F: GCAGTTTCAGAAAGCATCACGTGTG  R: CATGAACTGAGGGGACAGGATGTC |
| CD96-mCherry  3’ junction | 707 bp | F: CTACAACGTCAACATCAAGTTGGACATC  R: CCTCCTAAATTGTGGTAGTGTCTACTC |
| SFFV-FCGR3A  5’ junction | 815 bp | F: CGCTGGTGACTTGTTCTTCCAGGG  R: CTGGAAACATCTGATGGGTCTTAAGAAACTGC |
| SFFV-FCGR3A  3’ junction | 789 bp | F: GTTTCTTAAGACCCATCAGATGTTTCCAGG  R: CTGTGCAAACTCACAAATTAAGGGTACAGG |
| SFFV-CD226  5’ junction | 829 bp | F: ATGTCTGAGATCAGCAAGGAGGAAGAGGTG  R: CTGGAAACATCTGATGGGTCTTAAGAAACTGC |
| SFFV-CD226  3’ junction | 780 bp | F: GTTTCTTAAGACCCATCAGATGTTTCCAGG  R: GTCATGTTATTGGAAGCCATCGTTGAATTC |

| Primers for Round-the-horn PCR | | |
| --- | --- | --- |
| Junction | Amplicon | Primer (5’-3’) |
| SFFV-FCGR3A | 4235 bp | F: TGTTCTATGGTGGGGCTCCCTTGCCAG  R: GGACCAGGAATTAAAGAGCCTGGAGGCAAG |
| SFFV-CD226 | 4122 bp | F: TGGCCTTGCTTCATGTATACAGAGGTAAAGATGATC  R: GCAACAGGGTAGGATAATCCATGGTGGCCCCGTACCACC |
